# The nucleotide-binding oligomerization domain-containing protein 1 (NOD1) polymorphism S7N does not affect receptor function

**DOI:** 10.1186/1756-0500-7-124

**Published:** 2014-03-05

**Authors:** Sophie Mayle, Tom P Monie

**Affiliations:** 1 Department of Biochemistry, University of Cambridge, 80 Tennis Court Road, CB2 1GA, Cambridge, UK

**Keywords:** NOD1, NOD2, SNP, Polymorphism, Phosphorylation, RIG-I, NLR, Pattern recognition receptor, Serine, Caspase activation domain (CARD)

## Abstract

**Background:**

Activation and signal transduction in the Nucleotide binding, leucine-rich repeat containing receptor (NLR) family needs to be tightly regulated in order to control the inflammatory response to exogenous and endogenous danger signals. Phosphorylation is a common cellular mechanism of regulation that has recently been shown to be important in signalling in another family of cytoplasmic pattern recognition receptors, the RIG-I like receptors. In addition, single nucleotide polymorphisms can alter receptor activity, potentially leading to dysfunction and/or a predisposition to inflammatory barrier diseases.

**Findings:**

We have computationally analysed the N-terminus of NOD1 and found seven theoretical phosphorylation sites in, or immediately before, the NOD1 Caspase Activation Domain (CARD). Two of these, serine 7 and tyrosine 49 are also found as rare polymorphisms in the African-American population and European-American populations respectively. Mutating serine 7 to either an aspartic acid or an asparagine to mimic the potential impact of phosphorylation or the polymorphism respectively did not affect the response of NOD1 to ligand-mediated NFκB signalling.

**Conclusions:**

The NOD1 polymorphism S7N does not interfere with receptor function in response to ligand stimulation.

## Findings

### Background

Tight regulation and control of innate immune pattern recognition receptor (PRR) signalling pathways is essential to limit inappropriate receptor activation and excess inflammation. PRR signalling can be altered as a result of single nucleotide polymorphisms (SNPs). SNPs often serve as risk factors, or predispositions, for specific diseases. For example, in Nucleotide-binding Oligomerisation Domain (NOD) 2 the SNPs R702W, G908R, and 1007fsincC all inhibit receptor activation and predispose to Crohn’s Disease [1,2]. In contrast the mutations R334Q, R334W and L469F in the NOD region of NOD2 act as gain-of-function mutants and are associated with Blau syndrome and Early Onset Sarcoidosis [3-6]. Similarly, mutations in the NOD of the inflammasome forming protein NLRP3 result in hyper- or auto-activation of the receptor and lead to development of a spectrum of inflammatory diseases collectively known as cryopyrin associated periodic syndromes [7]. Polymorphisms in NOD1 have been less extensively studied but the E266K SNP has been linked to an increased risk of peptic ulceration in patients infected with *Helicobacter Pylori*[8].

Polymorphisms can affect protein function in unanticipated, and potentially unwelcome, ways. A more predictable and highly important mechanism of PRR regulation is post-translational modification. For example, serine 8 in helix 1 of the first RIG-I (Retinoic inducible gene –I) CARD and threonine 170 within helix 5 of the second RIG-I CARD are phosphorylated by protein kinase C alpha (PKCα) and PKCβ [9-12]. This restricts receptor activation by inhibiting the interaction of RIG-I with polyubiquitin and MAVS (mitochondrial antiviral signalling protein). The activity of MDA5 (melanoma differentiation-associated protein 5) is also influenced by phosphorylation. In this instance receptor function is inhibited by phosphorylation of serine 88 and serine 104; but stimulated when these residues are dephosphorylated by phosphoprotein phosphatase 1 α (PP1α) or PP1γ [13]. In the case of murine NLRC4 phosphorylation of S533 by PKCδ serves to enhance receptor activation [14].

Previous studies and ongoing work in our own laboratory have investigated the functional role of residues in the NOD1 CARD [15,16], but none have explicitly addressed phosphorylation. Given the importance of CARD phosphorylation in the regulation of RIG-I and MDA-5 signalling we hypothesised that serine 7 in human NOD1 could be a possible candidate for the regulation of NOD1 signalling and that the NOD1 SNP S7N may consequently disrupt receptor function.

## Methods

### Bioinformatics

The sequence of human NOD1 [Genbank: AAD28350.1] was submitted to the NetPhos 2.0 server [17] for the identification of potential serine, threonine and tyrosine phosphorylation sites. Solvent accessibility of residues in the monomeric [PDB: 2dbd] and dimeric [PDB: 2nz7] [18] forms of the NOD1 CARD was determined using ASAview [19]. Mammalian NOD1 orthologues were recovered from the NCBI non-redundant protein database using blastp (protein-protein BLAST) with human NOD1 [Genbank: AAD28350.1] as a search term using the standard parameters. Sequences were collated in FASTA format, aligned using MUSCLE [20] and incomplete or duplicate sequences manually removed. Thirty-six orthologues remained in the final alignment and the level of identity with human NOD1 residues 1-120 containing the CARD is shown in parentheses (all sequences were recovered from the NCBI REFSEQ database): Ailuropoda melanoleuca (84%) [REFSEQ: XP_002919315.1]; Bos taurus (83%) [REFSEQ: XP_598513.3]; Callithrix jacchus (95%) [REFSEQ: XP_002751479.1]; Canis lupus familiaris (86%) [REFSEQ: XP_539499.3]; Ceratotherium simum simum (88%) [REFSEQ: XP_004418924.1]; Condylura cristata (85%) [REFSEQ: XP_004677197.1]; Cricetulus griseus (83%) [REFSEQ: XP_003507840.1]; Dasypus novemcinctus (88%) [REFSEQ: XP_004453256.1]; Echinps telfairi (71%) [REFSEQ: XP_004702914.1]; Equus caballus (88%) [REFSEQ: XP_001499616.1]; Felis catus (88%) [REFSEQ: XP_003982953.1]; Heterocephalus_glaber (83%) [REFSEQ: EHB11938.1]; Homo Sapiens [REFSEQ: NP_006083.1]; Jaculus jaculus (78%) [REFSEQXP_004652693.1]; Loxodonta_africana (83%) [REFSEQ: XP_003407068.1]; Macaca fascicularis (98%) [REFSEQ: EHH52238.1]; Macaca mulatta (98%) [REFSEQ: EHH17407.1]; Mus musculus (79%) [REFSEQ: NP_766317.1]; Mustela putorius furo (84%) [REFSEQ: XP_004762622.1]; Nomascus leucogenys (99%) [REFSEQ: XP_003270528.1]; Octodon degus (86%) [REFSEQ: XP_004626575.1]; Odobenus rosmarus divergens (89%) [REFSEQ: XP_004414009.1]; Orcinus orca (82%) [REFSEQ: XP_004269993.1]; Ornithorhynchus anatinus (69%) [REFSEQ: XP_001512159.1]; Oryctolagus cuniculus (83%) [REFSEQ: XP_002713781.1]; Otolemur garnettii (84%) [REFSEQ: XP_003788597.1]; Ovis aries (80%) [REFSEQ: XP_004007979.1]; Pan troglodytes (100%) [REFSEQ: XP_001165528.1]; Pan paniscus (100%) [REFSEQ: XP_003833425.1]; Papio Anubis (98%) [REFSEQ: XP_003896196.1]; Pongo abelii (98%) [REFSEQ: XP_002818130.1]; Rattus norvegicus (78%) [REFSEQ: NP_001102706.1]; Saimiri boliviensis boliviensis (93%) [REFSEQ: XP_003935228.1]; Sorex araneus (67%) [REFSEQ: XP_004604307.1]; Sus scrofa (83%) [REFSEQ: BAG12313.1]; Trichechus manatus latirostris (82%) [REFSEQ: XP_004377558.1].

Consensus sequence images were generated using WebLogo v3.3 [21]. Molecular structure images were created using The PyMOL Molecular Graphics System, Version 1.5.0.5 Schrödinger, LLC. SNP frequencies were retrieved from the “Exome Variant Server” (http://evs.gs.washington.edu/EVS/).

### Plasmids

pUNO-NOD1 encodes full-length untagged human NOD1 and was a kind gift from Dr Peter Murray. Point mutants S7N, S7D and E56K were generated in the pUNO-NOD1 vector by site-directed mutagenesis. The plasmids pLuc and phrG (Promega) are components of the dual-luciferase assay and encode Firefly and Renilla luciferase respectively.

### NFκB-luciferase reporter assays

HEK293 cells were maintained in DMEM (Sigma) supplemented with 10% FCS, 100 μg/ml Penicillin/Streptomycin and 2 mM L-glutamine at 37°C and 5% CO_2_. Assays were performed in 96-well plates and 0.1 ng/well of the appropriate NOD1 plasmid was transfected using jetPEI (Polyplus Transfection) along with 1 ng/well of pLuc and phrG. Stimulation with up to 1000 ng/ml of ie-DAP (Invivogen), or 100 ng/ml of the control ligand iE-Lys (Invivogen) was performed simultaneously with transfection. 24 h later cells were lysed with 1 × passive lysis buffer (Promega) and luminescence measured with a FLUORstar Luminometer (BMG Labtech). Statistical analysis was performed on the data using an unpaired *t*-test with Welch’s correction for unequal variances. Protein expression was checked 24 h after transfection of HEK293 cells with 3 μg/DNA per well in a 6 well plate in the absence of stimulation. Proteins were visualised by western blot using the NOD1 monoclonal antibody 2A10 [22] and anti-GAPDH (Abcam: ab9485).

### Ethics statement

This work did not involve human subjects, human material or human data and therefore does not require ethical approval.

## Results and discussion

**Figure 1:**
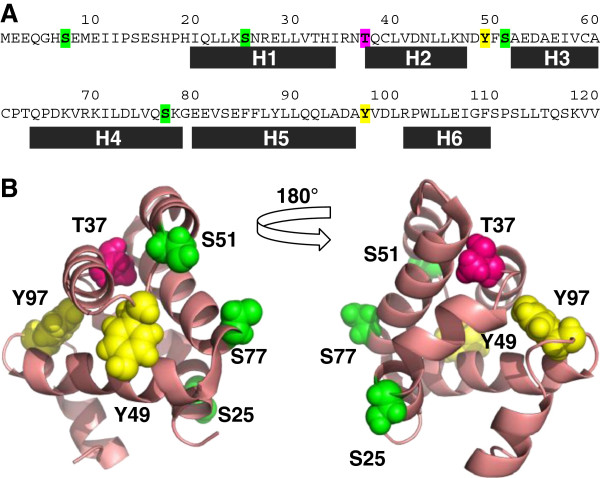
**Location of potential phosphorylation sites in the NOD1 CARD.** Potential serine, threonine and tyrosine phosphorylation sites in the first 120 residues of NOD1 were identified using NETPhos 2.0. **(A)** Location of potential phosphorylation sites in the primary sequence of the first 120 residues of NOD1. Black boxes denote the location of helices in the CARD. **(B)** Potential phosphorylation residues are shown as ball representations on the tertiary structure of the NOD1 CARD [PDB: 2dbd]. The left and right images are related by a 180° rotation around the vertical axis. Colouring in **(A)** and **(B)** is as follows: green – serine, pink – threonine, yellow - tyrosine.

Potential serine, threonine and tyrosine phosphorylation sites in and immediately adjacent to the CARD of the NLR family member NOD1 were identified using the prediction software NETPhos 2.0. Within the first 120 residues of human NOD1 (CARD structure 19-110) there were four predicted serine phosphorylation sites (residues 7, 25, 51, and 77); one predicted threonine phosphorylation site (residue 37); and two predicted tyrosine phosphorylation sites (residues 49 and 97) (Figure 1A). Analysis of the Exome Variant Server indicated that two of these residues are present as low frequency polymorphisms. Serine 7 is replaced with an asparagine in approximately 1 in 2200 of the African-American population; whilst tyrosine 49 is mutated to a cysteine in approximately 1 in 8500 of the European-American population.

We mapped the position of the potential phosphorylation sites onto the structure of the NOD1 CARD. Serine 7 is immediately prior to the structured CARD; serine 25 is within helix 1; threonine 37 is in the helix 1 – helix 2 loop; tyrosine 49 and serine 51 are in the helix 2 – helix 3 loop; serine 77 is in helix 4; and tyrosine 99 in the helix 5 – helix 6 loop (Figure 1B). The relative surface accessibility of these residues was determined using ASAview [19] from the NMR structure of the NOD1 CARD [PDB ID: 2dbd]. Serine 7 is not included in the structure so no information could be obtained for this residue. Its position near the start of the protein, prior to the first structured domain, makes it plausible that it is surface exposed; though the possibility remains that it may pack against the CARD helices in a manner that would inhibit kinase access. The order of relative surface accessibility of the remaining sites runs as follows: S77 > S25 > S51 > Y49 > Y97 > T37. As crystal structures of the NOD1 CARD show formation of a homodimer we also checked the relative surface accessibility of these residues in the dimeric form [PDB ID:2nz7]. The pattern of accessibility was broadly similar with the overall order S77 > S25 > S51 > Y49 > T37 > Y97. T37 and Y97 were consistently the two least accessible potential targets for phosphorylation in the whole CARD structure, making it highly unlikely that these residues undergo post-translational modification.

**Figure 2:**
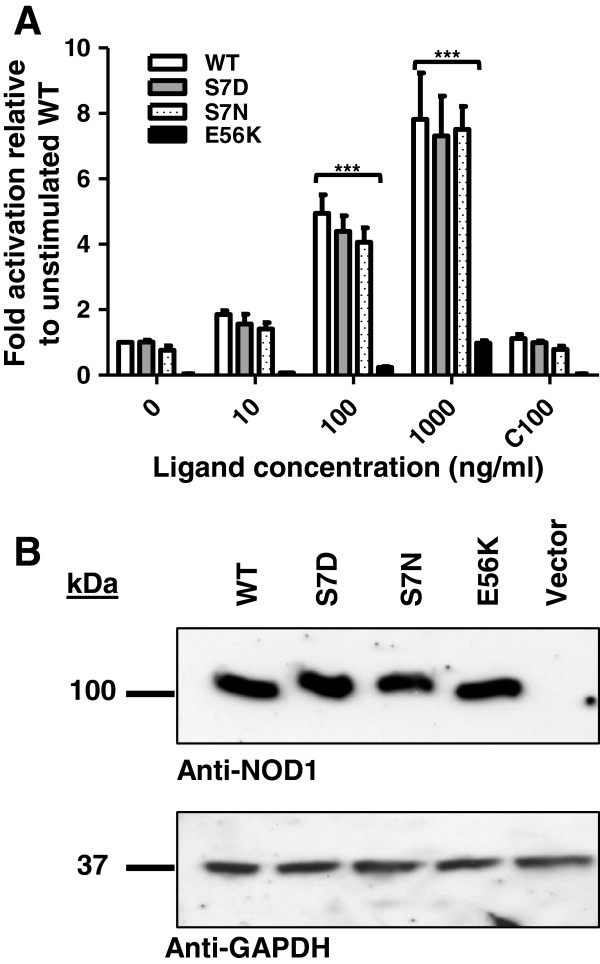
**Mutation of serine 7 does not affect NOD1 function. (A)** NFκB promoter-luciferase reporter assay of NOD1 pUNO wild type (WT), S7D, S7N and E56K in HEK293 cells. 0.1 ng/well DNA was stimulated with increasing doses of the ligand, iE-DAP, or 100 ng/ml of the inactive control ligand iE-Lys (C100). Cells were incubated for 24 hours before lysis. Results show the average of three independent experiments. *** = p < 0.0005. Error bars indicate SEM. **(B)** Immunoblots detecting expression of NOD1 pUNO WT, S7D, S7N, E56K and vector (pUNO) in HEK293 cells. Cells were transfected with 1.5 μg DNA and incubated for 24 hours in the absence of ligand and lysed and probed with the specified antibodies. Results are representative of three independent experiments.

Given the importance of serine phosphorylation in the regulation of RIG-I signalling and the potential for polymorphisms to disrupt receptor activity we mutated serine 7 to an asparagine to mimic the polymorphism; and to an aspartic acid to mimic phosphorylation. The ability of these mutants to activate NFκB signalling was then tested in HEK293 cells using a dual-luciferase NFκB reporter assay system. Both of the serine 7 mutants signalled in an identical manner to wild-type NOD1 in response to increasing concentrations of the stimulatory ligand diaminopimelic acid (iE-DAP). In contrast, the mutation E56K removed the ability of NOD1 to respond to ligand stimulation consistent with previous studies (p < 0.0005) [16] (Figure 2A). Western blot analysis confirmed that each construct expressed at a level comparable to wild-type protein (Figure 2B).

**Figure 3:**
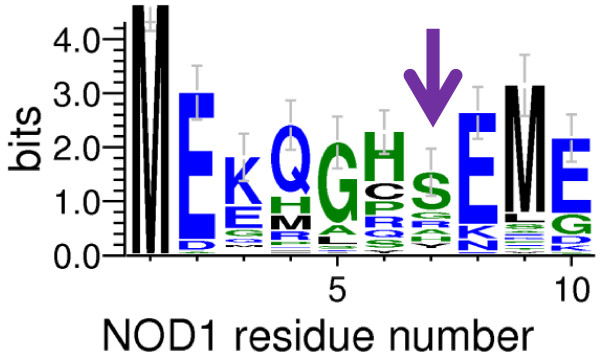
**Serine 7 shows limited conservation in mammalian NOD1.** A sequence logo representation of the first 10 amino acids of NOD1 in which letter height reflects the likelihood of finding a particular residue in that position. The location of serine 7 is marked with a purple arrow.

To reinforce the lack of impact on NOD1 function following mutation of serine 7 we compared the sequence conservation of this residue in NOD1 across 36 different mammalian species. Although serine was the most common residue in this position (21/36), numerous other residues were also present. These were: glycine (5/36), arginine (4/36), histidine (4/36), cysteine (1/36) and isoleucine (1/36) (Table 1 and Figure 3). The lack of extensive conservation in this position is consistent with a lack of role for phosphorylation of serine 7 in NOD1 function; and also with the lack of impact on receptor signalling of the S7N polymorphism. We also compared the cross-species conservation of the other putative phosphorylation sites (Table 1). Serine 25 and serine 51 were poorly conserved making it unlikely that they are important sites of phosphorylation. Threonine 37 was conserved in 34/36 species, whilst serine 77, tyrosine 49 and tyrosine 97 were all completely conserved. Combining the level of conservation with the relative surface accessibility suggests that serine 77 is a prime candidate for phosphorylation; that threonine 37 and tyrosine 97 play important structural roles; and that tyrosine 49 may be a potential phosphorylation target.

**Table 1:** Conservation of potential phosphorylation sites across 36 mammalian species

**Residue**	**Residue conservation**
Serine 7	21 serine, 5 glycine, 4 histidine, 4 arginine, 1 cysteine, 1 isoleucine
Serine 25	12 isoleucine, 10 serine, 9 valine, 4 threonine, 1 alanine
Threonine 37	34 threonine, 2 isoleucine
Tyrosine 49	36 tyrosine
Serine 51	19 alanine, 6 threonine, 5 serine, 3 glutamic acid, 1 isoleucine, 1 asparagine, 1 valine
Serine 77	36 serine
Tyrosine 97	36 tyrosine

This work has identified residues that may be potentially phosphorylated in the NOD1 CARD and which consequently have the potential to modulate receptor activity in a manner analogous to that seen with RIG-I and MDA-5. It also shows that mutation of one of these residues, serine 7, to either an aspartic acid to mimic phosphorylation, or to asparagine to reflect the natural polymorphism S7N does not affect receptor function. We can therefore conclude that the SNP S7N has no impact on NOD1 activation and signalling and that serine 7 is not involved in regulation of NOD1 signalling.

## Abbreviations

CARD: Caspase activation domain; MAVS: Mitochondrial antiviral signalling protein; MDA-5: Melanoma differentiation-associated protein 5; PKC: Protein kinase C; PRR: Pattern recognition receptor; NFκB: Nuclear factor kappa B; NLR: Nucleotide binding, leucine-rich repeat containing receptor; NLRC4: Nucleotide binding, leucine-rich repeat containing receptor, with a CARD 4; NLRP3: Nucleotide binding, leucine-rich repeat containing receptor with a pyrin 3; NOD1/2: Nucleotide oligomerisation domain containing 1/2; RIG-I: Retinoic acid inducible gene I; SNP: Single nucleotide polymorphism.

## Competing interests

The authors declare that they have no competing interests.

## Authors’ contributions

SM performed the reporter assays and western blots and edited the manuscript. TPM designed the experiments, performed the bioinformatic analyses, and wrote the manuscript. Both authors read and approved the final manuscript.
